# A Frontal Lobe Meningioma in a Child Leading to Visual Loss

**DOI:** 10.1155/2015/420964

**Published:** 2015-01-06

**Authors:** Nedime Sahinoglu-Keşkek, Gokhan Soker, Şakir Özgür Keşkek, Sehire Sahinoglu, Figen Unal, Fikret Unal, Selim Cevher

**Affiliations:** ^1^Department of Ophthalmology, Adana Numune Training and Research Hospital, 01240 Adana, Turkey; ^2^Department of Radiology, Adana Numune Training and Research Hospital, 01240 Adana, Turkey; ^3^Department of Internal Medicine, Adana Numune Training and Research Hospital, 01240 Adana, Turkey; ^4^Department of Radiology, Sisli Etfal Training and Research Hospital, 34360 Istanbul, Turkey

## Abstract

*Objective*. Meningiomas are benign primary meningeal tumors and are seen rare in children and adolescents. *Clinical Presentation and Intervention*. A 15-year-old Turkish boy reported a 1-month history of headache and blurred vision in both eyes. His visual acuity was 0.3 in both eyes with papilledema. Magnetic resonance imaging showed a 77 × 97 × 77 mm intracranial-extra-axial frontal lesion which compresses the chiasm. He was diagnosed with intracranial meningioma and referred to neurosurgery clinic. *Conclusion*. Ophthalmologists should be aware of the fact that papilledema and low vision can be caused by an intracranial tumor which compresses optic chiasm.

## 1. Introduction

Meningiomas are benign primary meningeal tumors and are seen rare in children and adolescents. These tumors arise from meninges, covering the brain and spinal cord, and therefore can occur in any number of locations. The diagnosis may be incidental or in response to a workup for ophthalmological or neurological symptoms. Even though they are slow-growing benign tumors, significant morbidity may result from compression of the oculomotor nerve, anterior visual pathways, or trigeminal nerve. Tumor expansion may lead to visual loss [1]. The purpose of this case report is to present a case of meningioma in a child associated with compressive optic neuropathy.

## 2. Case Report

**Figure 1:**
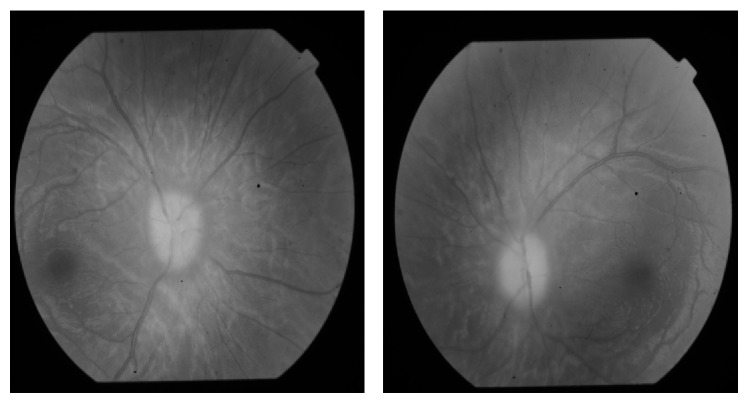
Funduscopic examination. Papilledema is seen on funduscopic examination.

**Figure 2:**
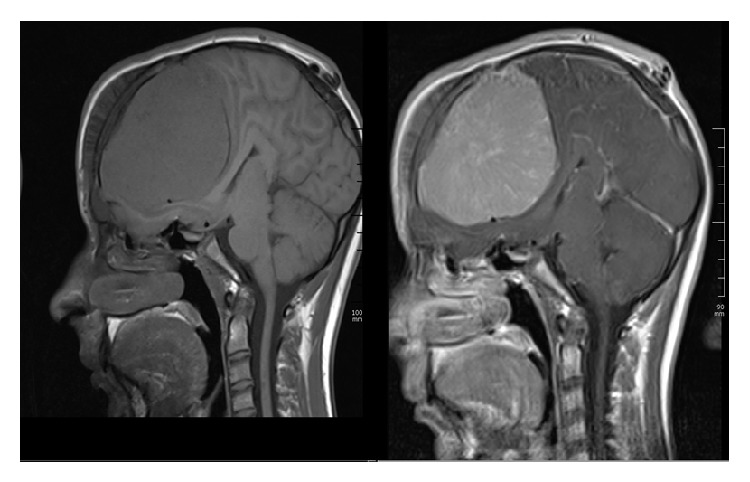
Magnetic resonance imaging. Contrast-enhanced sagittal T1-weighted MRI scan shows an extra-axial frontal tumoral lesion with intense enhancement.

**Figure 3:**
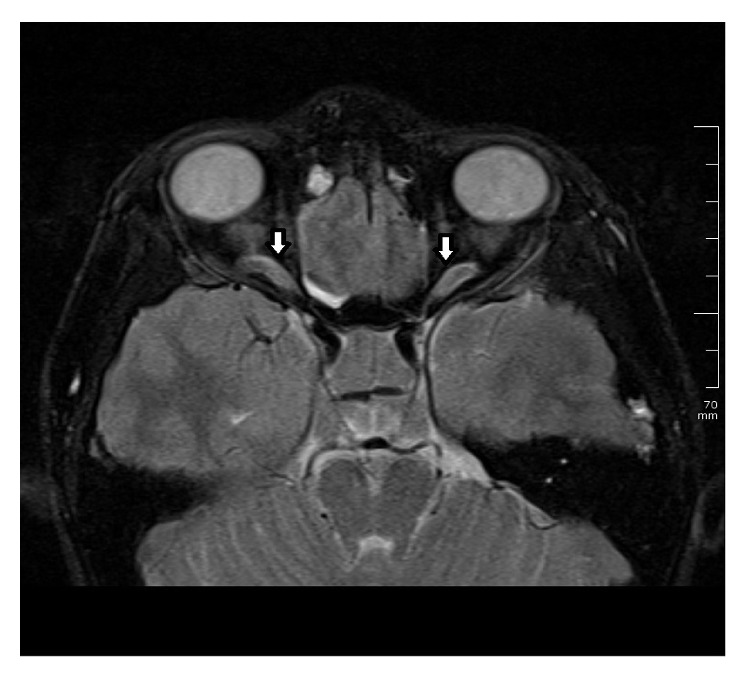
Magnetic resonance imaging. Contrast-enhanced axial T2-weighted MRI scan shows cerebrospinal fluid tracking along the optic nerve sheath.

**Figure 4:**
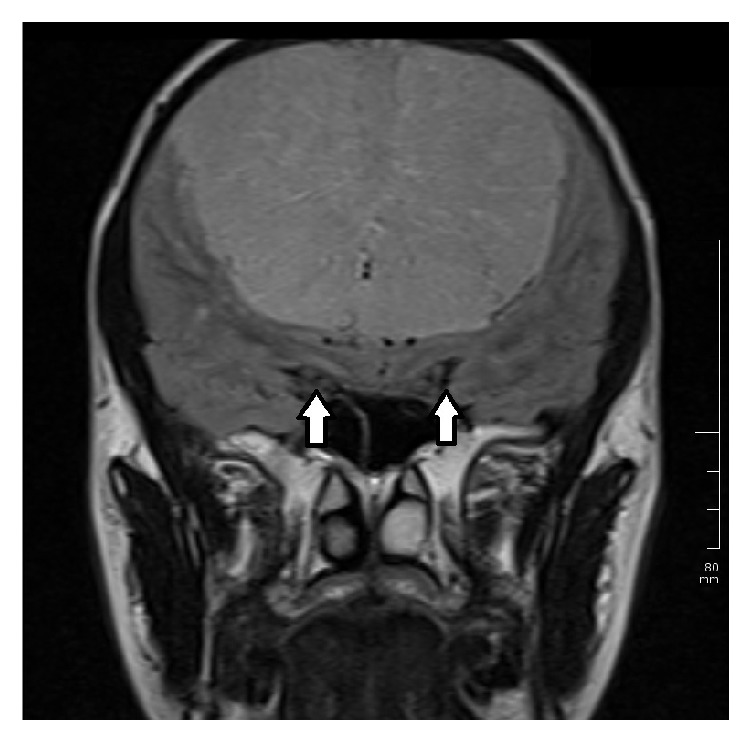
Magnetic resonance imaging. Coronal T2-weighted images show that the mass was exerting pressure on the chiasm.

**Figure 5:**
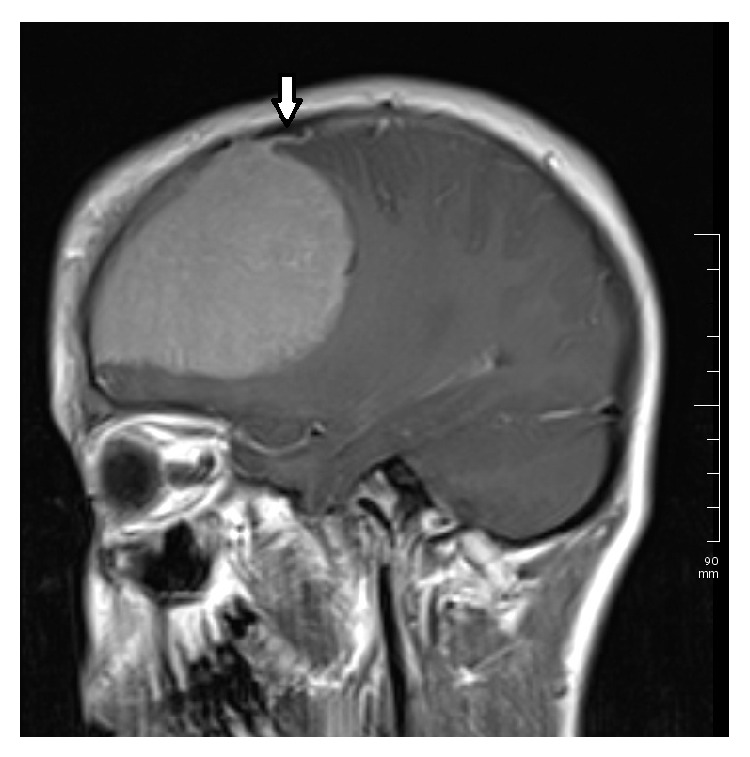
Magnetic resonance imaging. Sagittal T1-weighted MRI scan shows dural tail sign.

A healthy 15-year-old Turkish boy presented with one-month history of headache and visual impairment in both eyes. He visited the neurology department of a hospital one week before, but the results of general physical examination were unremarkable. It was suggested that he visit an ophthalmologist because of his visual disturbance. His best-corrected visual acuity (BCVA) was 0.3 in both eyes. There was papilledema on funduscopic examination (Figure 1). Magnetic resonance imaging (MRI) showed an extra-axial frontal tumoral lesion with intense enhancement (Figure 2). The dimensions of the lesion were measured as 77 × 97 × 77 mm and the lesion was located at superior aspect of the chiasm. MRI scan showed cerebrospinal fluid tracking along the optic nerve sheath which is the sign of increased intracranial pressure (Figure 3). Coronal T2-weighted images showed that the mass was also exerting pressure on the chiasm (Figure 4). Dural tail sign on sagittal T1-weighted images affirmed the diagnosis of meningioma (Figure 5). He was diagnosed with meningioma which compresses the normal brain tissue above the optic chiasm and referred to neurosurgery clinic. Operation was planned urgently. After gross-total resection, the patient progressed well with considerable improvement of his visual function. There has been no surgical or neurological complication recorded immediately after surgery.

## 3. Discussion

Meningiomas occur most commonly in the fifth decade of life with female preponderance, accounting for approximately 15–20% of primary intracranial tumors [2]. They are very rare in children with pediatric cases. Recently the Central Brain Tumor Registry of the United States reported that only 2.5% of all primary pediatric central nervous system tumors were meningiomas and in contrast to adult meningiomas, there is no female preponderance among pediatric cases [3, 4]. We presented a rare case of meningioma in a child with papilledema.

Frequent symptoms of meningioma are headache and visual disturbance [5]. Diagnosis of meningioma is made by a contrast-enhanced computed tomography (CT) or magnetic resonance imaging (MRI) scan. CT can be helpful in determining if the tumor invades the bone or if it is becoming hard like bone. Contrast-enhanced MRI well delineates the mass and its possible invasion into surrounding structures [6]. MRI characteristics of pediatric meningiomas are similar to adult meningiomas. They are usually isointense to hypointense on T1 and iso- to hyperintense on T2 and exhibit important enhancement [3]. The dural tail sign which was evident in our case is typically associated with meningioma. Dural tail sign is seen on contrast-enhanced magnetic resonance images as a thickening of the durameter that resembles a tail extending from a mass [7]. The existence of dural sign is not apparent in all cases of pediatric meningiomas that makes the radiological differential diagnosis with schwannomas difficult.

Aggressive surgical treatment is the treatment of choice in most cases which allows complete excision of the tumor in about 70–80% of cases. Kotecha et al. have stated that extent of initial surgical resection is also the strongest independent prognostic factor for children and adolescent meningiomas [8]. Hence, aggressive surgical management, to achieve gross-total resection, is the initial treatment of choice.

Most pediatric meningiomas are WHO grade I (80.6%) with WHO grade II accounting for 10.4% and grade III for 8.1% [9]. The pathological study of our case confirmed the diagnosis of WHO grade I meningioma.

With this case report we aimed to point out that papilledema and low vision can be caused by an intracranial tumor which compresses optic chiasm.
